# Sociodemographic, nutritional, and environmental factors are associated with cognitive performance among Orang Asli children in Malaysia

**DOI:** 10.1371/journal.pone.0219841

**Published:** 2019-07-15

**Authors:** Siti Fatihah Murtaza, Wan Ying Gan, Norhasmah Sulaiman, Zalilah Mohd Shariff, Siti Irma Fadhilah Ismail

**Affiliations:** 1 Department of Nutrition and Dietetics, Faculty of Medicine and Health Sciences, Universiti Putra Malaysia, Selangor, Malaysia; 2 Department of Psychiatry, Faculty of Medicine and Health Sciences, Universiti Putra Malaysia, Selangor, Malaysia; Pennsylvania State University, UNITED STATES

## Abstract

Children aged 2 to 6 years are in a crucial period of growth development, during which it is important for them to attain specific cognition related to concentration and attention so that they can perform well in school later in life. Various factors influence children’s cognition during this crucial period. However, to date, only a limited number of studies have examined the cognitive performance of underprivileged children living in poverty, particularly indigenous children (also known as Orang Asli children in Malaysia). Therefore, this cross-sectional study aimed to determine the associations between sociodemographic factors, nutritional factors (body composition and hemoglobin), and environmental factors (home environment and parasitic infections) with cognitive performance among Orang Asli children in Negeri Sembilan, Malaysia. The participants were 269 children (51% boys, 49% girls) aged 2 to 6 years (*M* = 4.0, *SD* = 1.2 years) and their mothers, from 14 Orang Asli villages. Face-to-face interviews were conducted with the mothers, and the children’s cognitive performance, operationalized as working memory index (WMI), processing speed index (PSI), and cognitive proficiency index (CPI), was assessed using the Wechsler Preschool and Primary Scale of Intelligence, Fourth Edition (WPPSI-IV). The children’s weight and height were measured, and their blood and stool samples were collected to assess hemoglobin level and parasitic infections, respectively. Multiple linear regression analysis showed that the father’s years of education (*β* = 0.262–0.342, *p* < 0.05), availability of learning materials at home (*β* = 0.263–0.425, *p* < 0.05), and responsiveness of the parent to the child (*β* = 0.192–0.331, *p* < 0.05) were consistently associated with all three cognitive indices (WMI, PSI, and CPI). A holistic approach involving parents, communities, and government agencies should be established to improve the cognitive performance of these underprivileged children.

## Introduction

An estimated 370 million people or more are classified as Indigenous or Aboriginal people worldwide [1]. In Malaysia, indigenous people are known as Orang Asli (OA), and they numbered approximately 4.4 million in 2015 [2]. Malaysia’s OA consist of 18 ethnic subgroups, which are divided into three major tribal groups: Semang (Negrito), Senoi, and Proto Malay (Aboriginal Malay). The OA commonly live in poor environments with an inadequate intake of energy, have a poor nutritional status, are exposed to high rates of infections such as those caused by parasites [3], and have specific deficiencies of nutrients including iron and iodine deficiencies [4,5].

Over the years, the Malaysian government has implemented various programs aimed primarily at improving the quality of life and general welfare of the OA. Examples of these programs include resettling, increasing income through cash-cropping and commercial activities, and physical support such as electricity, water supply, roads, houses, and foods [6]. However, little success has been achieved when OA are compared to other ethnic groups in Malaysia. Namely, the OA still face poverty as well as poor nutritional and health status, particularly where young children are concerned [6–8].

Cognition refers to the psychological process of memory, attention, learning, language, reasoning, and coordination of motor outputs [9]. Poverty, low socioeconomic status, poor health status, malnutrition, intestinal parasitic infections, poor home environment, low education level of parents, and micronutrient deficiencies are among the various factors that may contribute to children’s low cognitive performance [10–15]. These factors are common problems encountered by indigenous populations. A significant cognitive gap was found between indigenous and non-indigenous children due to the higher rate of poor nutritional and health status among indigenous children [16].

The preschool years represent a critical period for the development of mental processes that support an effective, goal-oriented approach to learning, particularly working memory and attention control [17]. These mental processes are often delayed in children growing up in poverty [18]. Research has shown that working memory and attention control lead to growth in emergent literacy and numeracy skills during the pre-kindergarten years [17]. Therefore, it is important to examine the cognitive performance of preschool children, especially indigenous children.

Children living in poverty usually experience fewer cognitive encouragements and enrichments in comparison to children from wealthier backgrounds. Children from low-income families frequently lack stimulation and the social skills necessary to prepare them for schooling [19]. For example, low-income parents interact less with their children and are minimally involved in their education due to unmanageable daily levels of stress [20]. Apart from a poor socioeconomic status, OA children in Malaysia have persistent problems of malnutrition, low birth weight, and poor iron status [21–23]. There is a concern that these types of health problems in OA children can negatively affect their cognition and academic achievement later on.

In Malaysia, no studies have been published on the cognitive performance of OA children. Several small-scale studies of OA children have focused on their body weight [8,23], dietary intake [7], parasitic infections [24], and food security [25]. Yet children need to have optimal cognitive development in order to prepare them for schooling. Hence, it is necessary to study the determining factors associated with OA children’s cognitive performance. Identifying factors associated with cognitive performance is crucial to improve health status, counter school dropout rates, and improve the academic performance and school attendance of OA children.

## Materials and method

### Study population and procedures

This cross-sectional study was conducted with OA children aged 2 to 6 years in the state of Negeri Sembilan, Malaysia. Two out of the seven districts in Negeri Sembilan, namely, Jempol (N = 16) and Kuala Pilah (N = 14), were purposely selected because they contain a high number of OA villages. The researchers approached every one of these villages (N = 30). Only 14 villages agreed to participate in the study. The reasons for not participating included not having a leader (*Tok Batin*) in the village and villagers who were culturally isolated and preferred not to mix with outsiders.

In the 14 participating villages, all OA children aged 2 to 6 years old were invited to take part in the study. Children who suffered from chronic illness and those with learning disabilities or developmental delays were excluded. Details of the sampling procedures have been described elsewhere [26]. The sample size was calculated by using G*Power 3.1.9.2 software for a multiple linear regression test. The total sample size needed was determined to be 234 participants, by assuming an effect size of 0.15, level of significance (α) of 0.05, power of 0.90, and expected response rate of 80%.

All instruments used in this study were translated into Malay by two experts from the fields of English and nutrition. The experts were fluent in both English and Malay. The instruments were also back-translated into English by another bilingual expert in nutrition. The English back-translation of the instruments was compared with the original English version and corrected as necessary. The instruments were then reviewed by expert panels from the fields of nutrition and psychology. After the review, the instruments were pre-tested prior to the data collection.

Before the study commenced, ethical clearance was sought from the Ethics Committee for Research Involving Human Subjects (JKEUPM) Universiti Putra Malaysia (Reference No.: FPSK(FR15)P001). Approval was also obtained from the Department of Orang Asli Development (JAKOA). Written informed consent was obtained from the mothers.

The researchers then conducted three visits to each OA village over a period of two weeks to one month. During the first visit, stool samples were collected from the children, and the mothers were interviewed at home using a Malay language questionnaire. On the second visit, the mothers and their children gathered in the village hall for blood sample collection and anthropometric measurements. A pediatrician conducted blood tests on the children. The cognitive performance test was administered to the children during the third visit, which took place at their home.

### Cognitive performance tests

The Wechsler Preschool and Primary Scale of Intelligence, Fourth Edition (WPPSI—IV) [27] was employed to measure the children’s cognitive performance using four selected tests: picture memory, zoo locations, bug search, and cancellation. The tests assessed the children’s working memory index (WMI) (picture memory and zoo location tests), processing speed index (PSI) (bug search and cancellation tests), and cognitive proficiency index (CPI) (picture memory, zoo location test, bug search, and cancellation tests). All three indices were measured on children aged 4 years and above. Only one index (WMI) was also measured on children under 4 years old. The tests are designed to measure the concentration and attention skills needed by children. Furthermore, the tests were deemed the most appropriate for children with limited educational opportunities living in a rural environment. These tests were non-verbal in that they required only action response from children.

A raw score was obtained for each test, and it was then adjusted to a scale score within the child’s own age group, using the standard age scale from the table provided in the WPPSI manual. The sum of the scale scores was converted to composite index scores for WMI, PSI, and CPI. The researcher was trained on the administration of the cognitive tests by one of the co-researchers in this study, a clinical psychologist. The tests were administered on each child by the researcher in a quiet room under the supervision of the clinical psychologist. Each child took approximately 30 minutes to complete the tests.

### Face-to-face interview

A Malay language questionnaire was used to obtain the participants’ sociodemographic information. The mothers provided information on their household (parents’ education level, parents’ monthly income, number of children, household size, and monthly household income) and children (age, sex, and birth order).

The Early Childhood Home Observation for Measurement of Environment (HOME) inventory for children aged 3 to 6 years old [28] is a semi-structured interview and observation tool used in this study. It is designed to assess parent-child interaction as well as the character and quantity of stimulation and support available to a child in the home environment. It consists of 55 items that are grouped into eight subscales: learning materials, language stimulation, physical environment, parental responsivity, academic stimulation, modeling of social maturity, variety in experience, and acceptance of the child. A binary-choice (yes/no) format was used to score the items. One point was awarded to items that were scored with “yes” and zero point to items that were scored with “no.” Each item was scored as a result of the observations of the researcher or the condition given by the mothers during a home visit. Higher total HOME scores indicate a high-quality home environment. In the present study, the Cronbach’s alpha coefficients of each subscale in the HOME ranged from 0.60 to 0.77, indicating a good internal consistency reliability.

### Anthropometric measurements

The children’s birth weight was collected from their health record books. Then, their current weight and height were measured according to standard procedures, using a TANITA Digital Weighing Scale HD-314 (TANITA Corporation, Arlington Heights, IL, USA) that rounded to the nearest 0.1 kg and a SECA body meter 206 (SECA, Hamburg, Germany) that rounded to the nearest 0.1 cm, respectively. Duplicate readings were obtained, and the average of the duplicate readings was recorded. The mean z-scores for Body Mass Index for age (BMI-for-age; BAZ), weight-for-age (WAZ), and height-for-age (HAZ) were determined using WHO AnthroPlus version 1.0.4 software (WHO, Geneva, Switzerland). The WHO Growth Reference 2007 [29] was used to classify the growth status of children aged five or older and the WHO Child Growth Standards 2006 [30] was used for children under five.

### Hemoglobin test

Blood sampling was carried out in the village hall a week after the questionnaire was completed, along with stool sampling. A pediatrician drew venous blood (3 mL) from each child to assess the hemoglobin (Hb) level using an EDTA tube. The laboratory analysis was outsourced to an accredited laboratory (BP Healthcare Group, Kuala Lumpur) using the three angled laser light scatter method (Abbott Ruby analyzer). The blood samples were kept in an icebox and safely transported to the nearest laboratory on the same day as the blood collection. Anemia was defined as having Hb concentration <11.0 g/dL for children under five and Hb concentration <11.5 g/dL for those aged five or older [31].

### Parasitic infections test

A thumb size sample of fresh stool for each of the children was collected in a stool container by their mothers. The mothers were instructed to wash their hands with soap and dry their hands before the stool collection. They used the stick provided by the researcher to collect the stool sample, ensuring that there was no urine, water, or sand in the sample. Once the stool was collected, the mothers contacted the researcher to collect it from their house. The stool samples were kept in an icebox and sent to an accredited laboratory (BP Healthcare Group, Kuala Lumpur) on the same day as the data collection to check for the presence of parasites, *Trichuris trichiuria* and *Ascaris lumbricoides*, using the direct microscopy (iodine) technique. The journey to the laboratory was approximately about 30 minutes to one hour.

### Statistical analysis

The data were analyzed using IBM SPSS Statistics 24.0 (IBM Corp., Armonk, NY, USA). All univariate analyses were carried out using descriptive statistics. Three separate multiple linear regression analyses were conducted on each cognitive performance index (WMI, PSI, and CPI) to determine their associated factors. Before performing linear regression analysis, the assumption of multicollinearity between variables was tested based on the variance inflation factor ≤ 10 [32]. No indication of multicollinearity was observed. All variables that had a value of *p* < 0.25 in the simple linear regression analysis were included in the backward multiple linear regression analysis. The criterion of *p* < 0.25 was based on the evidence that the threshold (*p* < 0.05) might exclude significant variables [33]. Backward multiple linear regression was used because it yielded results with a better R^2^ value and it had also been suggested for use in exploratory research to identify good predictors [33]. The level of statistical significance was set at *p* < 0.05.

## Results

Table 1 shows the characteristics of the OA children. A total of 269 children with a mean age of 4.04±1.21 years participated, out of 280 OA children aged 2 to 6 in the 14 villages. Approximately one third of the OA children (35%) had positive parasitic infections, in which 58% of them were infected by *Trichuris trichiuria*, 11% by *Ascaris lumbricoides*, and 36% by both parasites. Furthermore, 36% of the OA children were stunted, 28% were underweight, 6% were wasted/thin, and 22% were anemic.

**Table 1:** Characteristics of the participants (*n* = 269).

Characteristics	Mean ± SD / n (%)
**Sociodemographic factors**	
Child’s age (years)	4.04 ± 1.21
2	30 (11.2)
3	64 (23.8)
4	76 (28.3)
5	62 (23.0)
6	37 (13.8)
Sex	
Boy	137 (50.9)
Girl	132 (49.1)
Birth order	2.54 ± 1.69
Father’s age (years)	34.01 ± 7.38
Mother’s age (years)	30.59 ± 6.64
Years of education	
Father	6.17 ± 3.57
Mother	6.58 ± 3.72
Household size	5.74 ± 2.35
Number of children	2.97 ± 1.81
Monthly income of father (MYR)	500 (350, 900)^a^
Monthly income of mother (MYR)	350 (250, 500)^a^
Monthly household income (MYR)	700 (500, 1300)^a^
**Nutritional factors**	
Birth weight (kg)	2.83±0.56
Low birth weight (<2.5)	55 (20.4)
Normal birth weight	214 (79.6)
BMI-for-age z-score^b^	-0.43±1.05
BMI-for-age z-score^c^	-0.27±1.58
Wasting^d^/Thinness^e^ (<-2 SD)	16 (6.0)
Normal (≥-2SD to ≤+1SD)	224 (84.8)
Possible risk of overweight^d^ (>+1 SD)	5 (1.9)
Overweight (>+2 SD)	6 (2.3)
Obesity^d^/Severe obesity^e^ (>+3 SD)	13 (5.0)
Weight-for-age z-score	-1.26±1.27
Underweight (<-2 SD)	73 (27.7)
Normal	191 (72.3)
Height-for-age z-score	-1.56±0.97
Stunting (<-2 SD)	94 (35.6)
Normal	170 (64.4)
Hemoglobin level (g/dL)	11.81 ± 1.09
Anemia^f^	57 (21.7)
Normal	207 (78.3)
**Environmental factors**	
Parasitic infections (positive)	89 (35.0)
Home environment (0–55)	35.83 ± 5.96
Learning materials (0–11)	4.82 ± 2.23
Language stimulation (0–7)	6.51 ± 0.78
Physical environment (0–7)	4.54 ± 2.09
Responsivity (0–7)	5.01 ± 1.44
Academic stimulation (0–5)	3.67 ± 1.38
Modelling (0–5)	2.16 ± 1.23
Variety (0–9)	5.54 ± 1.29
Acceptance (0–4)	3.58 ± 0.60
**Cognitive performance**	
Working memory index	97.78 ± 15.62
Processing speed index	90.70 ± 17.34
Cognitive proficiency index	93.18 ± 17.35

^a^ Median (25^th^ percentile, 75^th^ percentile)

^b^ Children under five (*n* = 170)

^c^ Children aged five or older (*n* = 94)

^d^ Term used for children under five [30]

^e^ Term used for children aged five or older [29]

^f^ Anemia: hemoglobin concentration <11.0 g/dL for children under five and <11.5 g/dL for children aged five or older [31]

MYR = Ringgit Malaysia; USD 1 = MYR 4.14 as on 1 October 2018

Multiple linear regression results showed that higher WMI was significantly associated with more years of father’s education (*β* = 0.262, *p* = 0.007), higher father’s income (*β* = 0.358, *p* < 0.001), presence of parasites (*β* = -0.222, *p* = 0.026), more learning materials available at home (*β* = 0.263, *p* = 0.031), higher responsiveness of the parent to the child (*β* = 0.192, *p* = 0.049), and more variety in daily stimulation (*β* = 0.302, *p* = 0.007) (Table 2). These factors predicted 32% of the variance in WMI. The father’s income was the most significant predictor of WMI. In addition, findings of the separate analyses for WMI among OA children aged 2 to 3 years old and 4 to 6 years old are displayed at the Supplementary Material (S1 and S2 Tables).

**Table 2:** Simple linear regression and multiple linear regression results for the factors associated with Working Memory Index.

Characteristics	Simple linear regression						Multiple linear regression			
	Unstandardized coefficients	Standardized coefficients	95% CI		*p*-value	Unstandardized coefficients	Standardized coefficients	95% CI		*p*-value
	B	Beta	Lower bound	Upper bound		B	Beta	Lower bound	Upper bound	
**Sociodemographic Factors**										
Child’s age	1.897	0.149	0.354	3.440	0.016					
Father’s age	0.120	0.057	-0.143	0.382	0.370					
Mother’s age	0.139	0.060	-0.144	0.422	0.334					
Mother’s education	0.704	0.168	0.203	1.205	0.006					
Father’s education	0.915	0.208	0.384	1.445	0.001	1.056	0.262	0.300	1.812	0.007
Father’s income	0.003	0.109	0.000	0.007	0.084	0.015	0.358	0.007	0.023	<0.001
Mother’s income	0.704	0.168	0.203	1.205	0.006					
Birth order	-0.481	-0.052	-1.591	0.630	0.395					
**Nutritional Factors**										
Birth weight	3.388	0.122	0.053	6.724	0.047					
Weight-for-age	1.101	0.090	-0.385	2.587	0.146					
Height-for-age	3.141	0.195	1.203	5.078	0.002					
Hemoglobin level	1.453	0.102	-0.282	3.188	0.100					
**Environmental Factors**										
Parasitic infections	-9.697	-0.299	-13.555	-5.839	<0.001	-6.532	-0.222	-12.257	-0.807	0.026
Learning materials	1.478	0.224	0.653	2.303	<0.001	1.878	0.263	0.173	3.930	0.031
Language stimulation	4.063	0.217	1.713	6.413	0.001					
Physical environment	0.988	0.141	0.094	1.882	0.030					
Responsivity	1.877	0.184	0.590	3.164	0.004	1.927	0.192	0.100	3.854	0.049
Academic stimulation	2.755	0.258	1.428	4.083	<0.001					
Modelling	-0.632	-0.053	-2.158	0.893	0.415					
Variety	1.347	0.118	-0.107	2.801	0.069	3.204	0.302	5.504	0.903	0.007
Acceptance	0.505	0.021	-2.623	3.634	0.751					

Multiple linear regression model: R = 0.597, R^2^ = 0.357, Adjusted R^2^ = 0.318, F = 9.202, *p*<0.001

The father’s years of education (*β* = 0.342, *p* = 0.001), child’s hemoglobin level (*β* = 0.221, *p* = 0.031), availability of learning materials at home (*β* = 0.425, *p* = 0.002), and responsiveness of the parent to the child (*β* = 0.249, *p* = 0.016) had significant associations with PSI (Table 3). These factors predicted 40% of the variance in PSI. The most significant predictor of PSI was the availability of learning materials at home.

**Table 3:** Simple linear regression and multiple linear regression results for the factors associated with Processing Speed Index.

Characteristics	Simple linear regression						Multiple linear regression			
	Unstandardized coefficients	Standardized coefficients	95% CI		*p*-value	Unstandardized coefficients	Standardized coefficients	95% CI		*p*-value
	B	Beta	Lower bound	Upper bound		B	Beta	Lower bound	Upper bound	
**Sociodemographic Factors**										
Child’s age	0.242	0.127	-0.045	0.528	0.097					
Father’s age	-0.231	-0.094	-0.609	0.146	0.229					
Mother’s age	-0.033	-0.012	-0.436	0.370	0.872					
Mother’s education	2.717	0.197	0.687	4.748	0.009					
Father’s education	0.659	0.129	-0.123	1.440	0.098	1.550	0.342	0.608	2.493	0.001
Father’s income	0.002	0.061	-0.003	0.007	0.437					
Mother’s income	0.006	0.220	0.000	0.012	0.058					
Birth order	-1.859	-0.178	-3.405	-0.314	0.019					
**Nutritional Factors**										
Birth weight	2.333	0.079	-2.071	6.738	0.297					
Weight-for-age	0.785	0.059	-1.237	2.806	0.445					
Height-for-age	2.354	0.130	-0.363	5.072	0.089					
Hemoglobin level	2.106	0.135	-0.237	4.450	0.078	3.156	0.221	0.300	6.011	0.031
**Environmental Factors**										
Parasitic infections	-4.124	-0.114	-9.700	1.452	0.146					
Learning materials	2.563	0.331	1.463	3.664	<0.001	2.955	0.425	1.205	4.705	0.002
Language stimulation	6.801	0.312	3.685	9.917	<0.001					
Physical environment	1.027	0.123	-0.218	2.272	0.105					
Responsivity	2.825	0.228	1.006	4.643	0.003	3.276	0.249	0.625	5.927	0.016
Academic stimulation	4.929	0.358	2.993	6.866	<0.001					
Modelling	-1.036	-0.073	-3.162	1.091	0.338					
Variety	2.111	0.159	0.136	4.087	0.036					
Acceptance	-2.394	-0.085	-6.597	1.808	0.262					

Multiple linear regression model: R = 0.669, R^2^ = 0.447, Adjusted R^2^ = 0.399, F = 9.227, *p*<0.001

More years of the father’s education (*β* = 0.289, *p* = 0.007), higher father’s income (*β* = 0.191, *p* = 0.049), higher hemoglobin level (*β* = 0.237, *p* = 0.020), higher responsiveness of the parent to the child (*β* = 0.331, *p* = 0.002), more learning materials available at home (*β* = 0.400, *p* = 0.002), and more variety in daily stimulation (*β* = 0.327, *p* = 0.009) were significantly associated with CPI (Table 4). These factors predicted 42% of the variance in CPI. The availability of learning materials at home was the most significant predictor of CPI. Overall, the father’s years of education, availability of learning materials at home, and responsiveness of the parent to the child were the factors consistently associated with all three cognitive indices (WMI, PSI, and CPI).

**Table 4:** Simple linear regression and multiple linear regression results for the factors associated with Cognitive Proficiency Index.

Characteristics	Simple linear regression						Multiple linear regression			
	Unstandardized coefficients	Standardized coefficients	95% CI		*p*-value	Unstandardized coefficients	Standardized coefficients	95% CI		*p*-value
	B	Beta	Lower bound	Upper bound		B	Beta	Lower bound	Upper bound	
**Sociodemographic Factors**										
Child’s age	0.263	0.139	-0.024	0.549	0.072					
Father’s age	-0.040	-0.016	-0.422	0.342	0.837					
Mother’s age	0.139	0.052	-0.266	0.544	0.499					
Mother’s education	1.090	0.232	0.397	1.783	0.002					
Father’s education	0.799	0.155	0.011	1.586	0.047	1.363	0.289	0.392	2.334	0.007
Father’s income	0.004	0.117	-0.001	0.009	0.138	0.011	0.191	-0.001	0.023	0.049
Mother’s income	0.006	0.229	0.000	0.013	0.050					
Birth order	-1.302	-0.125	-2.871	0.267	0.103					
**Nutritional Factors**										
Birth weight	3.087	0.105	-1.336	7.511	0.170					
Weight-for-age	1.133	0.086	-0.882	3.147	0.269					
Height-for-age	3.055	0.170	0.353	5.757	0.027					
Hemoglobin level	2.372	0.153	0.037	4.707	0.047	3.458	0.237	0.578	6.338	0.020
**Environmental Factors**										
Parasitic infections	-8.780	-0.243	-14.266	-3.294	0.002					
Learning materials	2.160	0.278	1.029	3.292	<0.001	2.837	0.400	1.045	4.629	0.002
Language stimulation	6.112	0.280	2.943	9.282	<0.001					
Physical environment	1.106	0.131	-0.159	2.371	0.086					
Responsivity	2.588	0.208	0.742	4.434	0.006	4.444	0.331	1.725	7.163	0.002
Academic stimulation	4.350	0.313	2.353	6.346	<0.001					
Modelling	-1.608	-0.113	-3.754	0.538	0.141					
Variety	2.131	0.161	0.147	4.115	0.035	4.031	0.327	1.033	7.028	0.009
Acceptance	-0.863	-0.031	-5.107	3.382	0.689					

Multiple linear regression model: R = 0.693, R^2^ = 0.481, Adjusted R^2^ = 0.424, F = 8.481, *p*<0.001

## Discussion

This study shows that a father’s years of education and home environment, specifically the availability of learning materials at home and the responsiveness of the parent to the child, consistently appeared as the significant predictors for all three cognitive indices (WMI, PSI, CPI) for the OA children. To the best of our knowledge, this is the first study to demonstrate the associations of sociodemographic factors, nutritional factors, and environmental factors on cognitive performance in a sample of OA children aged 2 to 6 years old in Malaysia.

This study shows that more years of father’s education were associated with better working memory, processing speed, and cognitive proficiency performance for the children. These are all cognitive processes necessary for a child to prepare themselves prior to starting primary education. The findings accord with those of previous studies [34,35], where father’s education was associated with children’s cognitive performance. There is a possible explanation for the suggestion that a father’s level of education affects a child’s cognitive performance. It could be inferred that those fathers who had received higher education would have better job prospects, which in turn would increase the family’s income level and lead to their investment in better education and the health of their children [36]. In addition, higher education increases knowledge related to health, education, and parenting; for example, the father’s involvement in nurturing the children at an early age and having a positive attitude toward parenting were associated with better growth development in children [37]. Cabrera et al. [35] found that highly educated fathers spent more time with their children and were more supportive (characterized by emotional support and enthusiasm for the child’s autonomous work, responsiveness, and active attempts to expand the child’s knowledge and abilities), where these would improve children’s cognitive performance across ages. Furthermore, the results of a recent study showed that father-child time was strongly associated with children’s cognitive performance when said time was spent on educational activities (such as reading or educational play) [34]. These findings highlight the importance of the father’s involvement in a child’s cognitive performance. Thus, future interventions to improve the cognitive performance of OA children could promote positive parenting by fathers and encourage fathers to spend more time with their young children.

The present study also shows that higher father’s income was significantly associated with better working memory and cognitive proficiency in the OA children. This concurs with previous studies that found a positive correlation between income and children’s cognitive performance [37,38]. According to Dickerson and Popli [14], poor income families were limited in their ability to invest in their children’s education, which further decreased the children’s cognitive performance. Therefore, improving parents’ education levels and combating poverty are essential for the OA population to ensure parents have an adequate income and the knowledge to enable them to play an active role in their children’s education.

Beside sociodemographic factors, we found that the subscales from the HOME inventory contributed to the children’s cognitive performance, namely, the availability of learning materials at home, variety in daily stimulation, and parental responsiveness. These factors agree with several studies that also indicated that home environment had a significant correlation with children’s cognitive performance [15,39–42]. A longitudinal study of American children from birth to school age found that cognitive stimulation at home was more effective in increasing children’s learning abilities in their early childhood than the stimulation found in primary school [41]. Another study found that children with a poor quality of home environment had poorer cognitive performance than their counterparts who did not [42]. OA children are typically from a low socioeconomic background where they are less exposed to stimulating environments that promote cognitive performance.

Parents who provide learning materials at home, such as books or flashcards of letters and brain games that are either electronic or physical games, play the most significant role in improving the cognitive outcomes of their children, specifically their working memory and processing speed [38,43,44]. Meanwhile, parents’ responsive behaviors allow children to engage in more problem-solving skills involved in cognitive proficiency [45]. In general, the provision of a cognitively stimulating home environment during preschool years enhances a child’s cognitive development, for example, parents’ frequent reading to the child or helping the child with building blocks to learn letters and numbers [37]. Therefore, parents who are actively involved in stimulating children’s learning play a vital role in the improvement of their children’s cognitive performance. Combating poverty is important in the OA population to ensure that parents have adequate income and knowledge to provide a better home environment for their children and thus enhance their children’s cognitive performance.

It is well known that parasitic infections are among the neglected and highly prevalent tropical diseases among the OA in peninsular Malaysia [3,7]. In this study, one third of the OA children (35%) had parasitic infections. Further, parasitic infection was one of the significant predictors of the children’s working memory performance. These findings are consistent with several other studies that also found that parasitic infections impair children’s cognitive function and learning ability [12,46,47]. For example, Ezeamama et al. [46] showed significant associations of parasitic infections with learning ability, memory, and verbal fluency in children. Perignon et al. [12] also noted that parasitic infections were associated with poor working memory in children. A possible explanation of these results is that infected children are vulnerable to illness and frequently have diarrhea, stomach discomfort, and malabsorption of micronutrients, thus making it easy for them to lose their concentration and attention while doing the cognitive test [12,48].

Anemic children become tired easily and are hesitant, less playful, less attentive, and less willing to explore their environment [49]. This study found that Hb level significantly contributed to the processing speed and cognitive proficiency of the OA children, consistent with the results of Al-Mekhlafi et al. [10]. A longitudinal cohort study performed by Ai et al. [50] on 171 children from a developing region of China reported that children with low Hb levels had significantly lower scores on an IQ performance test. A cross-sectional study by Yang et al. [51] on 7331 children aged 4 to 6 years in 21 counties/cities in China found that anemic children were 1.3 times more likely to have poor scores in operational IQ and verbal IQ tests and 1.4 times more likely to have poor scores in full-scale IQ tests as compared to non-anemic children. Therefore, anemia is an important factor in children’s cognitive development.

Some study limitations deserve attention in future research. First, this was a cross-sectional study, and therefore causality between the factors associated with the children’s cognitive performance could not be well established. Cohort studies should be conducted in the future. Second, this study used Hb concentration as a proxy indicator of anemia and did not take into account serum ferritin and iron. Future studies should consider including serum ferritin and iron in order to identify the different types of anemia. Third, the total intelligence, Full-scale IQ (FSIQ) by WPPSI IV could not be calculated because not all of the WPPSI IV subtests were administered and it is a Western-based cognitive function test. Certain subtests that were most appropriate for children with limited educational opportunities living in a rural environment were chosen such as picture memory, zoo location, bug search, and cancellation tests, and these non-verbal tests were used to assess cognitive performance. Some items in the tests may be culturally biased. For example, a few pictures might not be familiar to the OA children such as bicycle helmet, hourglass, planet, and fire extinguisher. Furthermore, the composite index scores for WMI, PSI, and CPI in the WPPSI IV are based on the United States norm as no Malaysian norm is available. However, the WPPSI IV has been used in other Asian countries including Indonesia [39] and Pakistan [52]. Future studies should develop a Malaysian norm for the WPPSI IV. Fourth, the HOME scale has not been validated in Malaysia and may not truly reflect the culture of OA. However, this instrument has been widely used in developing countries [53] and is a useful measurement tool for factors that are correlated with cognitive performance in homes which stimulate thinking and learning [15,39]. Finally, the study sample may not represent OA children population in the state of Negeri Sembilan as there were only 14 out of 30 OA villages agreed to participate in this study. Despite these limitations, the current study provides insights into the factors that affect cognitive performance in the indigenous population, which could be translated into approaches to improve cognition among OA children.

## Conclusions

Our findings demonstrate that the father’s education level, father’s income, child’s Hb level, parasitic infections, and home environment, particularly the availability of learning materials, parental responsiveness to the child, and variety of daily stimulation were associated with the cognitive performance of OA children. Efforts to increase the education level of OA adults, improve the health and nutritional status of OA children, and increase cognitive stimulation at home should be emphasized in future interventions aimed at improving OA children’s cognitive performance. Moreover, appropriate early childhood interventions should be implemented to reduce the severity of the effects of poor cognitive performance; such interventions, in turn, could improve the readiness of OA children to learn in school and prevent dropouts.

## Supporting information

S1 File Dataset. Data used for the analyses presented in this paper.
(XLSX)

S2 File Questionnaire (English). The English questionnaire used in this paper.
(PDF)

S3 File Questionnaire (Malay). The Malay questionnaire used in this paper.
(PDF)

S1 Table Simple linear regression and multiple linear regression results for the factors associated with Working Memory Index for children aged 2 to 3 years old. Analysis for WMI in OA children aged 2 to 3 years old.
(DOCX)

S2 Table Simple linear regression and multiple linear regression results for the factors associated with Working Memory Index for children aged 4 to 6 years old. Analysis for WMI in OA children aged 4 to 6 years old.
(DOCX)

## References

[1] Gracey M, King M. Indigenous health part 1: determinants and disease patterns. Lancet. 2009;374(9683):65–75. 10.1016/S0140-6736(09)60914-4 19577695

[2] Hansen KB, Jepsen K, Jacquelin PL. The Indigenous World 2017. Copenhagen: The International Work Group for Indigenous Affairs (IWGIA); 2017.

[3] Elyana FN, Al-Mekhlafi HM, Ithoi I, Abdulsalam AM, Dawaki S, Nasr NA, et al A tale of two communities: Intestinal polyparasitism among Orang Asli and Malay communities in rural Terengganu, Malaysia. Parasit Vectors. 2016;9:398 10.1186/s13071-016-1678-z 27422533 PMC4947346

[4] Al-Mekhlafi HM, Al-Zabedi EM, Al-Maktari MT, Atroosh WM, Al-Delaimy AK, Moktar N, et al Effects of vitamin A supplementation on iron status indices and iron deficiency anaemia: a randomized controlled trial. Nutrients. 2014;6(1):190–206. 10.3390/nu6010190 PMC3916855 24384995

[5] Lim KK, Wong M, Wan Mohamud WN, Kamaruddin NA. Prevalence of iodine deficeincy disorder amongst Orang Asli in Hulu Selangor, Malaysia. Med Heal Sci J. 2012;11:2–6.

[6] Khor GL, Zalilah MS. The ecology of health and nutrition of “Orang Asli” (Indigenous People) women and children in Peninsular Malaysia. Tribes and Tribals. 2008;(2):66–77.

[7] Chua EY, Zalilah MS, Chin YS, Norhasmah S. Dietary diversity is associated with nutritional status of Orang Asli children in Krau Wildlife Reserve, Pahang. Malays J Nutr. 2012;18(1):1–13. 23713226

[8] Shashikala S, Kandiah M, Zalilah MS, Khor GL. Nutritional status of 1–3 years old children and maternal care behaviors in the Orang Asli of Malaysia. South African J Clin Nutr. 2005;18(2):173–180.

[9] Swaminathan S, Edward BS, Kurpad AV. Micronutrient deficiency and cognitive and physical performance in Indian children. Eur J Clin Nutr. 2013;67(5):467–474. 10.1038/ejcn.2013.14 23403875

[10] Al-Mekhlafi HM, Mahdy MA, Sallam AA, Ariffin WA, Al-Mekhlafi AM, Amran AA, Surin J. Nutritional and socio-economic determinants of cognitive function and educational achievement of Aboriginal schoolchildren in rural Malaysia. Br J Nutr. 2011;106(7):1100–1106. 10.1017/S0007114511001449 21492493

[11] Santos DN, Assis AMO, Bastos ACS, Santos LM, Santos CAS, Strina A, et al Determinants of cognitive function in childhood: A cohort study in a middle income context. BMC Public Health. 2008;8:202 10.1186/1471-2458-8-202 18534035 PMC2442073

[12] Perignon M, Fiorentino M, Kuong K, Burja K, Parker M, Sisokhom S, et al Stunting, poor iron status and parasite infection are significant risk factors for lower cognitive performance in Cambodian school-aged children. PLoS One. 2014;9(11):e112605 10.1371/journal.pone.0112605 25405764 PMC4236074

[13] Christensen DL, Schieve LA, Devine O, Drews-Botsch C. Socioeconomic status, child enrichment factors, and cognitive performance among preschool-age children: results from the Follow-Up of Growth and Development Experiences study. Res Dev Disabil. 2014;35(7):1789–1801. 10.1016/j.ridd.2014.02.003 24679548 PMC4997613

[14] Dickerson A, Popli G. Persistent poverty and children’s cognitive development: Evidence from the UK Millennium Cohort Study. J R Stat Soc. 2016;179:535–558.

[15] Warsito O, Khomsan A, Hernawati N, Anwar F. Relationship between nutritional status, psychosocial stimulation, and cognitive development in preschool children in Indonesia. Nutr Res Pract. 2012;6(5):451–457. 10.4162/nrp.2012.6.5.451 23198025 PMC3506877

[16] Wise S. Improving the early life outcomes of Indigenous children: implementing early childhood development at the local level Closing Gap Clear. Canberra: Australian Institute of Health and Welfare/Australian Institute of Family Studies; 2013.

[17] Welsh J, Nix RL, Blair C, Bierman KL, Nelson KE. The development of cognitive skills and gains in academic school readiness for children from low-income families. J Educ Psychol. 2011;102(1):43–53. 10.1037/a0016738 PMC2856933 20411025

[18] Noble KG, McCandliss BD, Farah MJ. Socioeconomic gradients predict individual differences in neurocognitive abilities. Dev Sci. 2007;10(4):464–480. 10.1111/j.1467-7687.2007.00600.x 17552936

[19] Ferguson HB, Bovaird S, Mueller MP. The impact of poverty on educational outcomes for children. Paediatr Child Health (Oxford). 2007;12(8):701–706. 10.1093/pch/12.8.701 PMC2528798 19030450

[20] Gratz J, Nation SO, Schools SO, Kurth-Schai R. The impact of parents’ background on their children’s education. Educ Stud. 2006;268:1–12.

[21] Al-Mekhlafi HM, Surin J, Atiya AS, Ariffin WA, Mahdy AKM, Abdullah HC. Anaemia and iron deficiency anaemia among aboriginal schoolchildren in rural Peninsular Malaysia: an update on a continuing problem. Trans R Soc Trop Med Hyg. 2008;102(10):1046–1052. 10.1016/j.trstmh.2008.05.012 18617209

[22] Khor GL, Misra S. Micronutrient interventions on cognitive performance of children aged 5–15 years in developing countries. Asia Pac J Clin Nutr. 2012;21(4):476–486. 23017305

[23] Wong CY, Zalilah MS, Chua EY, Norhasmah S, Chin YS, Siti Nur’Asyura A. Double-burden of malnutrition among the indigenous peoples (Orang Asli) of Peninsular Malaysia. BMC Public Health. 2015;15(1):680. 26194643 10.1186/s12889-015-2058-x PMC4508822

[24] Al-Delaimy AK, Al-Mekhlafi HM, Nasr NA, Sady H, Atroosh WM, Nashiry M, et al Epidemiology of intestinal polyparasitism among Orang Asli school children in rural Malaysia. PLoS Negl Trop Dis. 2014;8(8):e3074 10.1371/journal.pntd.0003074 25144662 PMC4140674

[25] Zalilah MS, Tham BL. Food security and child nutritional status among Orang Asli (Temuan) households in Hulu Langat, Selangor. Med J Malaysia. 2002;57(1):36–50. 14569716

[26] Siti Fatihah M, Gan WY, Norhasmah S, Zalilah MS. Factors associated with stunting among Orang Asli preschool children in Negeri Sembilan, Malaysia. Malays J Nutr. 2018;24(2):215–226.

[27] Weschler D, Scales PI. Wechsler Preschool and Primary Scale of Intelligence—Fourth Edition London: Pearson Education; 2012.

[28] Caldwell BM, Bradley RH. HOME inventory and administration manual. Little Rock, Ark: University of Arkansas for Medical Sciences; 2003.

[29] WHO. WHO Reference 2007: Growth reference data for 5–19 years. Geneva: World Health Organization; 2007.

[30] WHO Multicentre Growth Reference Study Group. WHO Child Growth Standards: Length/height-for-age, weight-for-age, weight-for-length, weight-for-height and body mass index-for-age: Methods and development. Geneva: World Health Organization; 2006.

[31] WHO. Serum ferritin concentrations for the assessment of iron status and iron deficiency in populations Vitamin and Mineral Nutrition Information System. Geneva: World Health Organization; 2011.

[32] Field A. Discovering statistics using IBM SPSS Statistics. 5th ed London, United Kingdom: Sage Publications Ltd.; 2017.

[33] Thayer JD. Stepwise regression as an exploratory data analysis procedure. New Orleans, LA: American Educational Research Association; 2002.

[34] Perales F, Baxter J. A matter of time: Father involvement and child cognitive outcomes. J Marriage Fam. 2019;81:164–184.

[35] Cabrera NJ, Shannon JD, Tamis-lemonda C. Fathers’ influence on their children’s cognitive and emotional development: From toddlers to pre-K. Appl Dev Sci. 2007;11(4):208–213.

[36] Lundborg P, Nilsson A, Rooth D-O. Parental education and offspring outcomes: Evidence from the Swedish compulsory schooling reform. Am Econ J. 2012;6(6570):253–278.

[37] Violato M, Petrou S, Gray R, Redshaw M. Family income and child cognitive and behavioural development in the United Kingdom: Does money matter? Health Econ. 2011;19(11):1300–1317. 10.1002/hec.1665 20945340

[38] Khanam R, Nghiem S. Family income and child cognitive and noncognitive development in Australia: Does money matter? Demography. 2016;53(3):597–621. 10.1007/s13524-016-0466-x 27083194

[39] Schneider N, Geiser E, Gosoniu LM, Wibowo Y, Gentile-Rapinett G, Tedjasaputra MS, et al A combined dietary and cognitive intervention in 3⁻5-year-old children in Indonesia: a randomized controlled trial. Nutrients. 2018;10(10):1394. 10.3390/nu10101394 PMC6213414 30275398

[40] MAL-ED Network Investigators. Early childhood cognitive development is affected by interactions among illness, diet, enteropathogens and the home environment: findings from the MAL-ED birth cohort study. BMJ Glob Health. 2018;3(4):e000752 10.1136/bmjgh-2018-000752 30058645 PMC6058175

[41] Crosnoe R, Leventhal T, Wirth RJ, Pierce KM, Pianta RC. Family socioeconomic status and consistent environmental stimulation in early childhood. Child Dev. 2010;81(3):972–987. 10.1111/j.1467-8624.2010.01446.x 20573117 PMC2892811

[42] Tong S, Baghurst P, Vimpani G, McMichael A. Socioeconomic position, maternal IQ, home environment, and cognitive development. J Pediatr. 2007;151(3):284–288. 10.1016/j.jpeds.2007.03.020 17719939

[43] Demoulin C, Kolinsky R. Does learning to read shape verbal working memory? Psychon Bull Rev. 2016;703–722. 10.3758/s13423-015-0956-7 26438254

[44] Nouchi R, Taki Y, Takeuchi H, Hashizume H, Nozawa T. Brain training game boosts executive functions, working memory and processing speed in the young adults: A randomized controlled trial. PLoS One. 2013;8(2):e55518 10.1371/journal.pone.0055518 23405164 PMC3566110

[45] Merz EC, Landry SH, Johnson UY, Williams JM. Effects of a responsiveness-focused intervention in family child care homes on children’s executive function. Early Child Res Q. 2017;34:128–139. 10.1016/j.ecresq.2015.10.003 PMC4770831 26941476

[46] Ezeamama AE, Friedman JF, Acosta LP, Bellinger DC, Langdon GC, Manalo DL, et al Helminth infection and cognitive impairment among Filipino children. Am J Trop Med Hyg. 2005;72(5):540–548. 15891127 PMC1382476

[47] Jasti A, Ojha SC, Singh YI. Mental and behavioral effects of parasitic infections: A review. Nepal Med Coll J. 2007;9(1):50–56. 17593680

[48] Hall A, Hewitt G, Tuffrey V, De Silva N. A review and meta-analysis of the impact of intestinal worms on child growth and nutrition. Matern Child Nutr. 2008;4(SUPPL.1):118–236. 18289159 10.1111/j.1740-8709.2007.00127.x PMC6860651

[49] Ngure FM, Reid BM, Humphrey JH, Mbuya MN, Pelto G, Stoltzfus RJ. Water, sanitation, and hygiene (WASH), environmental enteropathy, nutrition, and early child development: Making the links. Ann N Y Acad Sci. 2014;1308(1):118–128. 24571214 10.1111/nyas.12330

[50] Ai Y, Zhao SR, Zhou G, Ma X, Liu J. Hemoglobin status associated with performance IQ but not verbal IQ in Chinese preschool children. Pediatr Int. 2012;54(5):669–675. 10.1111/j.1442-200X.2012.03648.x 22507306 PMC3404215

[51] Yang L, Liu JM, Ye RW, Hong SX, Zheng JC, Ren AG. Correlation on hemoglobin concentration and the development of cognition among pre-school children. Zhonghua Liu Xing Bing Xue Za Zhi. 2010;31(4):389–393. 20513281

[52] Gilani I. Cognitive potential and its predictors in children from a rural district of Pakistan. J Ayub Med Coll Abbottabad. 2017;29(1):68–73. 28712178

[53] Jones PC, Pendergast LL, Schaefer BA, Rasheed M, Svensen E, Scharf R, et al Measuring home environments across cultures: Invariance of the HOME scale across eight international sites from the MAL-ED study. J Sch Psychol. 2017;64:109–127. 10.1016/j.jsp.2017.06.001 28735604 PMC5540057

