# Supplementary material for: Sociodemographic, nutritional, and environmental factors are associated with cognitive performance among Orang Asli children in Malaysia
Source: PLoS One. 2019 Jul 15;14(7):e0219841. doi: 10.1371/journal.pone.0219841 (PMC6629085; doi:10.1371/journal.pone.0219841)
Supplement: S2 File — The English questionnaire used in this paper. (PDF) [file pone.0219841.s002.pdf]

Reference No.: _____________

Part A1: Child's Background

1. Date of birth: ____(dd)_____(mm)_______(yyyy)

2. Sex: [   ] Male   [   ] Female

3. Birth weight (from health record book): _______ grams

4. Birth order: ________

Part A2: Family's Background

No.	Sub-ethnic<br><br>Code (1)	Sex<br><br>Code (2)	Relationship with family<br><br>Code (3)	Date of birth	Marital status<br><br>Code (4)	Highest education level	
						Code (5)	No. of years
1							
2							
3							
4							
5							
6							
7							
8							
9							
10							
11							
12							
13							
14							
15							

Code (1): Sub-ethnic

Temuan = 1
Temiar = 2
Jah hut = 3
Semelai = 4
Lain-lain = 5
____

Code (2): Sex

Male = 1
Female = 2

Code (3): Relationship with family

Head of family = 1
Husband/wife = 2
Child = 3
Father = 4
Mother = 5
Father-in-law = 6
Mother-in-law = 7
Brother = 8
Sister = 9
Younger brother/sister = 10
Step child = 11
Foster child = 12
Grandchild = 13
Nephew/niece = 14

Code (4): Marital status

Single = 1
Married = 2
Widowed = 3
Divorced = 4

Code (5): Education level

Primary education =1
Lower secondary education =2
Upper secondary education =3
Diploma/*Maktab* =4
No formal education =5
Not yet schooling =6
Kindergarten =7

Family size : ______________
No. of children : ______________
No. of children attending school : _____________

Part A3: Household's Income and Employment

No.	Relationship with family Code (3)	Main Job		Side Job		Monthly income (RM)	
		Type (Please specify)	Level of employment Code (6)	Types (Please specify)	Level of employment Code (6)	Main	Side
1							
2							
3							
4							
5							
6							
7							
8							
9							
10							
11							
12							
13							
14							
15							

Code (6): Level of employment

Government sector	=1	Pension	=4
Private sector	=2	Unemployed (not including disabled and senior citizen)	=5
Self-employed	=3	House wife	=6

Part B:
Place a plus (+) or minus (-) in the box alongside each item depending on whether the behavior is observed during the visit, or if the parent reports that the conditions or events are characteristic of the home environment. Enter the subtotals and the total on the Summary Sheet. ***Observation (O), Either (E), or Interview (I) is indicated for each item.***

**I. LEARNING MATERIAL**		**II. LANGUAGE STIMULATION**	
1. Child has toys which teach colors, sizes, and shapes. **E**		12. Child has toys that help teach names of animals. **E**	
2. Child has 3 or more puzzles. **E**		13. Child is encouraged to learn the alphabet. **I**	
3. Child has a record, tape, or CD player and at least 5 children's records, tapes, or CDs. **E**		14. Parent teaches child simple verbal manners (please, thank you, I'm sorry). **I**	
4. Child has toys or games permitting free expression. **E**		15. Parent encourages child to talk and takes time to listen. **I**	
5. Child has toys or games requiring refined movements. **E**		16. Child is permitted choice in breakfast or lunch menu. **I**	
6. Child has toys or games which help teach numbers. **E**		17. Parent uses correct grammar and pronunciation. **O**	
7. Child has at least 10 children's books. **E**		18. Parent's voice conveys positive feelings about child. **O**	
8. At least 10 books are visible in the apartment or home. **E**		**III. PHYSICAL ENVIRONMENT**	
9. Family buys and reads a daily newspaper. **I**		19. Building appears safe and free of hazards. **O**	
10. Family subscribes to at least one magazine. **I**		20. Outside play environment appears safe. **O**	
11. Child is encouraged to learn shapes. **I**		21. Interior of home or apartment is not dark or perceptually monotonous. **O**	
22. Neighborhood is aesthetically pleasing. **O**		**V. ACADEMIC STIMULATION**	
23. House has 100 square feet of living space per person. **O**		33. Child is encouraged to learn colors. **I**	
24. Rooms are not overcrowded with furniture. **O**		34. Child is encouraged to learn patterned speech. **I**	
25. House is reasonably clean and minimally cluttered. **O**		35. Child is encouraged to learn spatial relationships. **I**	
**IV. RESPONSIVITY**		36. Child is encouraged to learn numbers. **I**	
26. Parent holds child close 10-15 minutes per day. **I**		37. Child is encouraged to learn to read a few words. **I**	
27. Parent converses with child at least twice during visit. **O**		**VI. MODELING**	
28. Parent answers child's questions or requests verbally. **O**		38. Some delay of food gratification is expected. **I**	
29. Parent usually responds verbally to child's speech. **O**		39. TV is used judiciously. **I**	
30. Parent praises child's qualities twice during visit. **O**		40. Child can express negative feelings without harsh reprisal. **I**	
31. Parent caresses, kisses, or cuddles child during visit. **O**			
32. Parent helps child demonstrate some achievement during visit. **O**		41. Child can hit parent without harsh reprisal. **I**	
42. Parent introduces Visitor to child. **O**		**VIII. ACCEPTANCE**	

VII. VARIETY		52. No more than one instance of physical punishment occurred during the past week. **I**	
43. Child has real or toy musical instrument. **E**		53. Parent does not scold or yell at or derogate child more than once. **O**	
44. Child is taken on outing by a family member at least every other week. **I**		54. Parent does not use physical restraint during visit. **O**	
45. Child has been on a trip more than 50 miles during past year. **I**		55. Parent neither slaps nor spanks child during visit. **O**	
46. Child has been taken to a museum during past year. **I**			
47. Parent encourages child to put away toys without help. **I**			
48. Child eats at least one meal on most days with mother and father. **I**			
49. Parent lets child choose certain favorite food products or brands at grocery store. **I**			
50. Parent uses complex sentence structure and vocabulary. **O**			
51. Child's art work is displayed some place in house. **O**			
TOTAL:<br>I _______ II _______ III _______ IV _______ V _______ VI _______ VII _______ VIII _______ TOTAL _________			

Part C: Anthropometric measurements

Types of measurement	First reading	Second reading	Average
Height (cm)			
Weight (kg)			

Part D: Biochemical tests

No.	Test	Reading
1.	**Hemoglobin (g/dL)**	
2.	**Stool (epg)**	
	a) Ascaris lumbricoides	
	b) Trichuris trichiuria	

End

THANK YOU
