# Supplementary material for: Sociodemographic, nutritional, and environmental factors are associated with cognitive performance among Orang Asli children in Malaysia
Source: PLoS One. 2019 Jul 15;14(7):e0219841. doi: 10.1371/journal.pone.0219841 (PMC6629085; doi:10.1371/journal.pone.0219841)
Supplement: S3 File — The Malay questionnaire used in this paper. (PDF) [file pone.0219841.s003.pdf]

No. Rujukan: _______

Bahagian A1: Latar belakang anak

1. Tarikh lahir anak: ____(hh)_____(bb)_______(tttt)

2. Jantina: [   ] Lelaki   [   ] Perempuan

3. Berat lahir anak (buku rekod kesihatan): _______ gram

4. Turutan anak: ________

Bahagian A2: Latar Belakang Keluarga

Bil.	Nama	Suku<br><br>Kod (1)	Jantina<br><br>Kod (2)	Hubungan dengan keluarga<br><br>Kod (3)	Tarikh lahir	Taraf perkahwinan<br><br>Kod (4)	Tahap pendidikan paling tinggi	
							Kelulusan<br><br>Kod (5)	Bil. tahun
1								
2								
3								
4								
5								
6								
7								
8								
9								
10								
11								
12								
13								
14								
15								

Kod (1): Suku

Temuan = 1
Temiar = 2
Jah hut = 3
Semelai = 4
Lain-lain = 5
____

Kod (2): Jantina

Lelaki = 1
Perempuan = 2

Kod (3): Hubungan ahli keluarga dengan ketua isirumah

Ketua = 1
Suami/Isteri = 2
Anak = 3
Bapa = 4
Ibu = 5
Bapa mertua= 6
Ibu mertua= 7
Abang = 8
Kakak = 9
Adik = 10
Anak tiri = 11
Anak angkat = 12
Cucu = 13
Anak saudara = 14

Kod (4): Taraf perkahwinan

Bujang = 1
Berkahwin = 2
Balu = 3
Bercerai = 4

Kod (5): Tahap pendidikan

Sekolah rendah =1
Sekolah menengah rendah=2
Sekolah menengah tinggi =3
Diploma/Maktab =4
Sekolah pondok =5
Tidak bersekolah =6
Belum bersekolah =7
Tadika =8

Bil. Ahli Keluarga: ______________ Bil. Anak Bersekolah : _____________
Bil. Anak : ______________

Bahagian A3: Pendapatan dan Pekerjaan Ahli Isi Rumah

Bil.	Hubungan dengan keluarga Kod (3)	Pekerjaan Utama		Pekerjaan sampingan		Pendapatan pada bulan lepas (RM sebulan)	
		Jenis (nyatakan)	Taraf Kod (6)	Jenis (nyatakan)	Taraf Kod (6)	Utama	Sampingan
1							
2							
3							
4							
5							
6							
7							
8							
9							
10							
11							
12							
13							
14							
15							

Kod (6): Taraf pekerjaan

Pekerja sektor kerajaan	=1	Pesara	=4
Pekerja sektor swasta	=2	Penganggur (tidak termasuk orang tua dan orang cacat)	=5
Pekerja sendiri	=3	Suri rumah tangga	=6

Bahagian B: Penjagaan Psikososial Anak

Sila tandakan (+) atau (-) di petak berkenaan bagi setiap soalan sekiranya tingkahlaku tersebut dikesan ketika lawatan atau ibubapa melaporkan keadaan tersebut merupakan ciri-ciri persekitaran biasa di rumah. *Pemerhatian (O), mana-mana (E) atau temubual (I) seperti yang ditunjukkan untuk setiap item.*

**I. BAHAN PEMBELAJARAN**		**II. RANGSANGAN BAHASA**	
1. Kanak-kanak (KK) mempunyai permainan yang membantu KK mengenal warna, saiz dan bentuk. **E**		12. KK mempunyai permainan yang mengajar nama haiwan. **E**	
2. KK mempunyai tiga atau lebih *puzzles*. **E**		13. Ibubapa menggalakkan KK untuk mengenal huruf. **I**	
3. KK mempunyai CD pemain rekod atau pita perakam dan sekurang-kurangnya mempunyai 5 rekod, pita atau CD. **E**		14. Ibubapa mengajar KK cara lisan yang mudah (tolong, terima kasih, minta maaf). **I**	
4. KK mempunyai permainan yang mengeluarkan ekspresi bebas. **E**		15. Ibu bapa menggalakkan KK bercakap dan mengambil masa untuk mendengar. **I**	
5. KK mempunyai permainan yang memerlukan pergerakan halus. **E**		16. KK dibenarkan memilih menu sarapan atau makan tengahari. **I**	
6. KK mempunyai permainan yang membantu KK mengenal nombor. **E**		17. Ibubapa menggunakan tatabahasa dan sebutan yang betul. **O**	
7. KK mempunyai sekurang-kurangnya 10 buah buku KK. **E**		18. Suara ibubapa menyampaikan perasaan positif mengenai KK. **O**	
8. Sekurang-kurangnya 10 buah buku dapat dilihat di dalam rumah. **E**		**III. PERSEKITARAN FIZIKAL**	
9. Keluarga membeli dan membaca surat khabar harian. **I**		19. Bangunan kelihatan selamat dan bebas bahaya. **O**	
10. Keluarga melanggan sekurang-kurangnya 1 buah majalah. **I**		20. Persekitaran di luar rumah kelihatan selamat untuk bermain. **O**	
11. KK digalakkan untuk belajar kepelbagaian jenis bentuk. **I**		21. Hiasan dalaman rumah tidak gelap atau membosankan. **O**	
22.Kejiranan kelihatan menyenangkan. **O**		**V. RANGSANGAN AKADEMIK**	
23. Keluasan rumah adalah 100 kaki persegi untuk ruang hidup seorang. **O**		33. KK digalakkan untuk mengenal warna. **I**	
24. Bilik adalah tidak terlalu sempit dengan perabot. **O**		34. KK digalakkan untuk belajar ucapan bercorak. **I**	
25. Rumah kelihatan bersih dan hanya sedikit bersepah. **O**		35. KK digalakkan untuk belajar hubungan *spatial*. **I**	
**IV.TINDAKBALAS**		36.KK digalakkan untuk belajar nombor. **I**	
26. Ibubapa memegang anak dengan rapat 10-15 min sehari. **I**		37. KK digalakkan untuk belajar membaca beberapa perkataan. **I**	

27. Ibubapa bercakap dengan KK sekurang-kurangnya 2 kali ketika lawatan. **O**		**VI. PEMODELAN**	
28. Ibubapa menjawab soalan atau permintaan KK secara verbal. **O**		38. Kelambatan penyuapan makanan adalah dijangka. **I**	
29. Ibubapa selalu bertindakbalas secara verbal terhadap percakapan KK. **O**		39. TV digunakan secara bijaksana. **I**	
30. Ibubapa memuji kualiti KK 2 kali ketika lawatan. **O**		40. KK boleh meluahkan perasaan negatif tanpa tindak balas yang keras. **I**	
31. Ibubapa menjaga, mencium atau memeluk KK ketika lawatan. **O**			
32. Ibubapa membantu KK menunjukkan beberapa pencapaian ketika lawatan. **O**		41. KK boleh memukul ibubapa tanpa tindak balas yang keras. **I**	
42. Ibubapa mengenalkan pelawat kepada KK. **O**		**VIII. PENERIMAAN**	
**VII. PELBAGAI**		52. Tidak lebih daripada satu contoh hukuman fizikal berlaku pada minggu lepas. **I**	
43. KK mempunyai alat muzik yang sebenar atau mainan. **E**		53. Ibubapa tidak memarahi atau menjerit kepada KK lebih daripada sekali ketika lawatan. **O**	
44. KK dibawa berjalan-jalan oleh ahli keluarga sekurang-kurangnya selang seminggu. **I**		54. Ibubapa tidak menggunakan halangan fizikal ketika lawatan. **O**	
45. KK pernah berada dalam perjalanan lebih daripada 50 batu (80km) pada tahun lepas. **I**		55. Ibubapa tidak menampar atau merotan KK ketika lawatan. **O**	
46. KK pernah dibawa ke muzium pada tahun lepas. **I**			
47. Ibubapa menggalakkan KK untuk mengemas permainan selepas bermain tanpa bantuan. **I**			
48. KK makan sekurang-kurangnya sekali sehari bersama ibu (atau penjaga perempuan) dan ayah (atau penjaga lelaki). **I**			
49. Ibubapa membenarkan KK memilih produk atau jenama makanan kegemaran di kedai runcit. **I**			
50. Ibubapa menggunakan struktur ayat dan tatabahasa yang kompleks. **O**			
51. Hasil lukisan KK dipamerkan di suatu tempat di rumah. **O**			
JUMLAH:<br>I ________ II _______ III ________ IV ________ V _______ VI _______ VII _______ VIII _______ JUMLAH _____			

Bahagian C: Ukuran antropometrik anak

Jenis ukuran	Ukuran 1	Ukuran 2	Purata
Tinggi (cm)			
Berat (kg)			

Bahagian D: Ujian biokimia

Bil.	Ujian	Bacaan
1.	**Hemoglobin (g/dL)**	
2.	**Tahi (epg)**	
	a) Ascaris lumbricoides	
	b) Trichuris trichiuria	

SOALAN TAMAT

TERIMA KASIH
