# Supplementary material for: Sociodemographic, nutritional, and environmental factors are associated with cognitive performance among Orang Asli children in Malaysia
Source: PLoS One. 2019 Jul 15;14(7):e0219841. doi: 10.1371/journal.pone.0219841 (PMC6629085; doi:10.1371/journal.pone.0219841)
Supplement: S1 Table — Analysis for WMI in OA children aged 2 to 3 years old. (DOCX) [file pone.0219841.s004.docx]

**S1 Table. Simple linear regression and multiple linear regression results for the factors associated with Working Memory Index for children aged 2 to 3 years old**

| **Characteristics** |  | |  | | | **Simple regression** | | | | | | | | | | **Multiple regression** | | | | | | | |
| --- | --- | --- | --- | --- | --- | --- | --- | --- | --- | --- | --- | --- | --- | --- | --- | --- | --- | --- | --- | --- | --- | --- | --- |
|  | **Unstandardized coefficients** | | | **Standardized coefficients** | | | **95% CI** | | | | ***p*-value** | | **Unstandardized coefficients** | | **Standardized coefficients** | | | **95% CI** | | | | ***p*-value** | |
|  | **B** | | | **Beta** | | | **Lower bound** | | | **Upper bound** |  | | **B** | | **Beta** | | | **Lower bound** | | **Upper bound** | |  | |
| **Sociodemographic Factors** | | | | | | | |  |  | |  |  | |  | | |  | |  | |  | |  |
| Father’s age | | 0.112 | | | 0.072 | | | -0.219 | 0.443 | | 0.504 |  | |  | | |  | |  | |  | |  |
| Mother’s age | | 0.311 | | | 0.177 | | | -0.051 | 0.672 | | 0.091 |  | |  | | |  | |  | |  | |  |
| Mother’s education | | 0.251 | | | 0.077 | | | -0.427 | 0.929 | | 0.463 |  | |  | | |  | |  | |  | |  |
| Father’s education | | 0.841 | | | 0.324 | | | 0.198 | 1.484 | | 0.011 | 0.181 | | -0.022 | | | 1.470 | | 0.724 | | 0.047 | |  |
| Father’s income | | 0.001 | | | 0.027 | | | -0.004 | 0.005 | | 0.080 | 0.445 | | 0.004 | | | 0.028 | | 0.016 | | 0.013 | |  |
| Mother’s income | | -0.003 | | | -0.086 | | | -0.015 | 0.009 | | 0.636 |  | |  | | |  | |  | |  | |  |
| Birth order | | 0.572 | | | 0.086 | | | -0.824 | 1.969 | | 0.417 |  | |  | | |  | |  | |  | |  |
| **Nutritional Factors** | |  | | |  | | |  |  | |  |  | |  | | |  | |  | |  | |  |
| Birth weight | | 6.292 | | | 0.268 | | | 1.548 | 11.036 | | 0.010 |  | |  | | |  | |  | |  | |  |
| Weight-for-age | | -0.498 | | | -0.051 | | | -2.577 | 1.581 | | 0.635 |  | |  | | |  | |  | |  | |  |
| Height-for-age | | 2.261 | | | 0.182 | | | -0.322 | 4.843 | | 0.085 |  | |  | | |  | |  | |  | |  |
| Hemoglobin level | | -0.116 | | | -0.010 | | | -2.596 | 2.363 | | 0.926 |  | |  | | |  | |  | |  | |  |
| **Environmental Factors** | | | | |  | | |  |  | |  |  | |  | | |  | |  | |  | |  |
| Parasitic infections | | -7.093 | | | -0.288 | | | -12.156 | -2.029 | | 0.007 |  | |  | | |  | |  | |  | |  |
| Learning materials | | 1.493 | | | 0.275 | | | 0.401 | 2.585 | | 0.008 | 0.738 | | 1.401 | | | 5.591 | | 3.496 | | 0.003 | |  |
| Language stimulation | | 1.394 | | | 0.101 | | | -1.473 | 4.261 | | 0.337 |  | |  | | |  | |  | |  | |  |
| Physical environment | | 0.987 | | | 0.169 | | | -0.220 | 2.195 | | 0.108 |  | |  | | |  | |  | |  | |  |
| Responsivity | | 1.791 | | | 0.231 | | | 0.211 | 3.371 | | 0.027 |  | |  | | |  | |  | |  | |  |
| Academic stimulation | | 1.331 | | | 0.132 | | | -0.756 | 3.419 | | 0.208 |  | |  | | |  | |  | |  | |  |
| Modelling | | 0.670 | | | 0.072 | | | -1.260 | 2.599 | | 0.492 |  | |  | | |  | |  | |  | |  |
| Variety | | 2.001 | | | 0.190 | | | -0.159 | 4.160 | | 0.069 | 0.740 | | 2.215 | | | 10.854 | | 6.535 | | 0.005 | |  |
| Acceptance | | 2.883 | | | 0.136 | | | -1.529 | 7.295 | | 0.198 |  | |  | | |  | |  | |  | |  |

Multiple linear regression model: R=0.515, R^2^=0.266, Adjusted R^2^=0.228, F=7.057, *p*<0.001
