# Supplementary material for: Sociodemographic, nutritional, and environmental factors are associated with cognitive performance among Orang Asli children in Malaysia
Source: PLoS One. 2019 Jul 15;14(7):e0219841. doi: 10.1371/journal.pone.0219841 (PMC6629085; doi:10.1371/journal.pone.0219841)
Supplement: S2 Table — Analysis for WMI in OA children aged 4 to 6 years old. (DOCX) [file pone.0219841.s005.docx]

**S2 Table. Simple linear regression and multiple linear regression results for the factors associated with Working Memory Index for children aged 4 to 6 years old**

| **Characteristics** |  | |  | | | **Simple regression** | | | | | | | | | | **Multiple regression** | | | | | | | |
| --- | --- | --- | --- | --- | --- | --- | --- | --- | --- | --- | --- | --- | --- | --- | --- | --- | --- | --- | --- | --- | --- | --- | --- |
|  | **Unstandardized coefficients** | | | **Standardized coefficients** | | | **95% CI** | | | | ***p*-value** | | **Unstandardized coefficients** | | **Standardized coefficients** | | | **95% CI** | | | | ***p*-value** | |
|  | **B** | | | **Beta** | | | **Lower bound** | | | **Upper bound** |  | | **B** | | **Beta** | | | **Lower bound** | | **Upper bound** | |  | |
| **Sociodemographic Factors** | | | | | | | |  |  | |  |  | |  | | |  | |  | |  | |  |
| Father’s age | | 0.057 | | | 0.026 | | | -0.283 | 0.397 | | 0.742 |  | |  | | |  | |  | |  | |  |
| Mother’s age | | -0.062 | | | -0.026 | | | -0.426 | 0.301 | | 0.735 |  | |  | | |  | |  | |  | |  |
| Mother’s education | | 0.943 | | | 0.224 | | | 0.326 | 1.560 | | 0.003 |  | |  | | |  | |  | |  | |  |
| Father’s education | | 0.733 | | | 0.159 | | | 0.032 | 1.434 | | 0.041 | 0.935 | | 0.217 | | | 0.079 | | 1.790 | | 0.033 | |  |
| Father’s income | | 0.004 | | | 0.002 | | | 0.000 | 0.009 | | 0.060 | 0.018 | | 0.411 | | | 0.009 | | 0.027 | | <0.001 | |  |
| Mother’s income | | 0.004 | | | 0.163 | | | -0.002 | 0.010 | | 0.163 |  | |  | | |  | |  | |  | |  |
| Birth order | | -1.155 | | | -0.122 | | | -2.574 | 0.265 | | 0.110 |  | |  | | |  | |  | |  | |  |
| **Nutritional Factors** | |  | | |  | | |  |  | |  |  | |  | | |  | |  | |  | |  |
| Birth weight | | 2.102 | | | 0.079 | | | -1.908 | 6.111 | | 0.302 |  | |  | | |  | |  | |  | |  |
| Weight-for-age | | 1.540 | | | 0.128 | | | -0.267 | 3.347 | | 0.094 |  | |  | | |  | |  | |  | |  |
| Height-for-age | | 3.239 | | | 0.199 | | | 0.819 | 5.658 | | 0.009 |  | |  | | |  | |  | |  | |  |
| Hemoglobin level | | 1.908 | | | 0.136 | | | -0.202 | 4.018 | | 0.076 |  | |  | | |  | |  | |  | |  |
| **Environmental Factors** | | | | |  | | |  |  | |  |  | |  | | |  | |  | |  | |  |
| Parasitic infections | | -9.849 | | | -0.302 | | | -14.681 | -5.017 | | <0.001 | -9.541 | | -0.295 | | | -16.585 | | -2.496 | | 0.009 | |  |
| Learning materials | | 1.575 | | | 0.524 | | | 0.541 | 2.609 | | 0.003 | 1.573 | | 0.238 | | | -0.128 | | 3.275 | | 0.047 | |  |
| Language stimulation | | 4.868 | | | 1.467 | | | 1.972 | 7.763 | | 0.001 |  | |  | | |  | |  | |  | |  |
| Physical environment | | 0.754 | | | 0.099 | | | -0.383 | 1.891 | | 0.192 |  | |  | | |  | |  | |  | |  |
| Responsivity | | 2.362 | | | 0.210 | | | 0.710 | 4.015 | | 0.005 | 2.990 | | 0.244 | | | 0.527 | | 5.454 | | 0.018 | |  |
| Academic stimulation | | 3.426 | | | 0.268 | | | 1.576 | 5.277 | | <0.001 |  | |  | | |  | |  | |  | |  |
| Modelling | | -0.549 | | | -0.043 | | | -2.486 | 1.387 | | 0.576 |  | |  | | |  | |  | |  | |  |
| Variety | | 1.284 | | | 0.106 | | | -0.529 | 3.097 | | 0.164 | 5.114 | | 0.445 | | | 2.382 | | 7.846 | | <0.001 | |  |
| Acceptance | | 0.033 | | | 0.001 | | | -3.813 | 3.879 | | 0.986 |  | |  | | |  | |  | |  | |  |

Multiple linear regression model: R=0.689, R^2^=0.475, Adjusted R^2^=0.419, F=8.446, *p*<0.001
